# Supplementary material for: Measuring information flux between social media and stock prices with Transfer Entropy
Source: PLoS One. 2021 Sep 23;16(9):e0257686. doi: 10.1371/journal.pone.0257686 (PMC8459996; doi:10.1371/journal.pone.0257686)
Supplement: Attachment — Submitted filename: Response to reviewers.pdf [file pone.0257686.s002.pdf]

List of Responses:

1) Reviewer #1's observation: *"- How is the polarity defined and calculated? Apparently, it is only the sum of the polarities of the words in a sentence. Is that so? The meaning can change notably by changing the ordering of the words. Are some specific constructions recognized?"*

Response: In lines 198-214 we expand the definition of the algorithm and how its logic is structured, considering the inefficiency when words are not correctly ordered. The source code was added into the 3rd footnote at the end of page 7.

2) Reviewer #1's observation: *"- Please check the definition of Y_t. Currently its states "For a given day t, the Polarity of the Day Y was constructed by adding ...". Probably Y refers to the company. Similarly, improve the definition of X_t."*

Response: In lines 248-255 were corrected and improved according to the feedback from the reviewer.

3) Reviewer #1's observation: *"- Equation (5) is the core of the analysis performed. Thus, it should be clearly explained. What are the indices k and l? Also, what are x_t^{(k)} and y_t^{(l)}? How are the transition probabilities calculated?"*

Response: In lines 271-275 we introduced the definitions of k and l, furthermore the explanation of why the combinations of k=l=3 was chosen for presenting the results were introduced in lines 359-363.

4) Reviewer #1's observation: *"- The comment in the first paragraph in pg. 8 is unclear. The data (both from Twitter and stock prices) are sampled daily, so that a discrete set is obtained. Why is this a problem for the application of Eq. (5) which also refers to discrete datasets?"*

Response: In lines 296-300 we introduce the configuration of the model to data discretization in which a number of bins is selected in order to partition the data, since the time series X and Y have the characteristic of being continuous. In lines 358-359 the quantiles selected to discretize the data was presented.

5) Reviewer #1's observation: *"- The authors report a negative Pearson correlation coefficient between companies and indexes (last line of the first paragraph in pg. 9). Where is this shown? One cannot notice any red spots in Fig. 3."*

Response: In lines 307-308 we correct the interpretation of the graph in which there's little to no correlation between the stock prices and indexes. Furthermore, in the Figure 3 the explanation was improved by mentioning this correct interpretation.

6) Reviewer #1's observation: *"- In tables 2, 3 and 4, a p value is given. How is this calculated? What does it imply? It is not mentioned in the "Methodology section"*

Response: In lines 321-324, it is introduced how the p -value is calculated for the null hypothesis for no information exchange for the simulated distribution that corresponds for the transfer entropy estimations.

7) Reviewer #1's observation: *"- What evidence is given to support that the negative news has a greater impact in prices than positive ones? This goes unnoticed in the main text but concluded."*

Response: The first comment regarding negative news with greater impact that positive news is introduced in line 59. Further evidence is introduced in line 418 and presented Figure 2 and Figure 7 which visually helps with the interpretation that there is concordance with the cited article *"Wesley S. Chan. Stock price reaction to news and no-news: drift and reversal after headlines. Journal of Financial Economics. 2001; 18:203–260"* . There is also mentioned that when the companies that presented information flow from Sentiment Index towards the Stock Price are plotted in parallel it can be appreciated the aforementioned effect.

In line 445 *t*he conclusion was adjusted to present a precedent for further investigation given the evidence presented in the introduction and explained further in the Discussion section.

Reviewer #2: In the article, the authors offer a contribution to answering the question that, as well expressed by them, is “How can we translate this impact in a quantitative method and in a statistically measurable fashion that can be scientifically replicated?”

A very thorough introduction substantiates the claim of the authors that there is an important relation between news, or the comments of experts, on the way stock prices move. The way the authors describe the Language Processing algorithm is very enlightening, and results were obtained in a series of different ways, making them more robust.

Figure 5 is THE figure with the major explanation capability and impact in the article.

A suggestion for future work would be doing this in moving windows, so as to add a dimension of time to the analysis being made.

1) Reviewer #2’s observation: *“1. I think some of the captions should be enlarge. A caption should be understood without the need to resort to the main text. Particularly, for Figure 1, Figure 3.”*

Response: Figure’s captions were enlarged according to the authors’ suggestion, in which in Figure 1 we explain the steps involved in the generalized framework followed to get to our model results. In Figure 3 we improved the correlation matrix graph and corrected the explanation.

2) Reviewer #2’s observation: **“***In equation (3), the use of the difference between stock prices and not the return, which would be divided by the price of the day before, does not introduce problems with stationarity of the time series? This is issue demands some explanation, as it will be questioned by many readers.”*

Response: In line 368 we introduce the interest in working with stationary version of the Stock price, this to change most of the literature review that prefer to work with the stock performance. To demonstrate that it would not present issues, stationary tests were performed as explained in lines 270-375 in which all tests confirmed that the time series satisfied the statistical requirements.

3) Reviewer #2’s observation: **“***Why are the authors using k=l=3 for Transfer Entropy? Maybe it would be good to elaborate on it.”*

Response: The definition of how k and l are calculated are introduced in lines 271-275 and equation 6 was added, in lines 364-368 we explain why k=l=3 was selected and what

would be the interpretation in the results. Meaning that the impact of the sentiment index would have its maximum effect in a 3-day span.

4) Reviewer #2's observation: *"In the Conclusion, the authors write: "The asymmetric theory of information states that the negative news have a greater impact in stock prices than positive news. In this study we present evidence to support this theory." It was not at all clear to me where the results that were presented substantiate this important claim, as I haven't seen a separate analysis for positive and negative sentiments."*

Response: The first comment regarding negative news with greater impact that positive news is introduced in line 59. Further evidence is introduced in line 418 and presented Figure 2 and Figure 7 which visually helps with the interpretation that there is concordance with the cited article *"Wesley S. Chan. Stock price reaction to news and no-news: drift and reversal after headlines. Journal of Financial Economics. 2001; 18:203–260"* . There is also mentioned that when the companies that presented information flow from Sentiment Index towards the Stock Price are plotted in parallel it can be appreciated the aforementioned effect.

In line 445 *t*he conclusion was adjusted to present a precedent for further investigation given the evidence presented in the introduction and explained further in the Discussion section.

5) Reviewer #2's observation: Suggestions for grammar or orthography.
Please change, at the author's discretion:
"Farag and Cressy [29], studied how price limits, aimed to prevent speculation 11 amongst traders when new information is released in the market." The paragraph is nuclear.

• "in a random matter" to "in a random manner".
• "according the information" to "according to when the information".
• "market movements that coincides" to "market movements that coincide".
• "of 2018. Which" to "of 2018, which".
• "etc), this step" to "etc); this step".
• Please correct punctuation in displayed equations.
• "Even so, provide" to "Even so, it provides".
• "statistical analysis the combination" to "statistical analysis of the combination".
• "For measuring the signal transfer" to "In order to measure the signal transfer".
• "in **different stock markets, we" to "in different stock markets. We".**
• **"information flow**. Compared" to "   ".
• There are plenty others. I recommend a thorough review on writing.

Response: The grammar was reviewed according as requested

5) Reviewer #2's observation: "I will suggest to the authors to compare their findings with the ones obtained by other authors. This will help to show the contribution of this research, which is not clear in my opinion. In the introduction section, authors write about the EMF, the MDH and the SIAH. At this point, I will suggest commenting on the main aspects of the Adaptive Market Hypothesis and the Fractal Market Hypothesis."

Response: Comparisons to other 2 models were presented in lines 440-452. In addition, it was expressed that the major contribution from this research is the comparison of a company's index versus other companies and that there is communication between multiple companies affected by multiple Sentiment Indexes in lines 465-470. In the introduction the AMH and FMH were added as suggested, in lines 11-32.
