# Supplementary material for: Measuring information flux between social media and stock prices with Transfer Entropy
Source: PLoS One. 2021 Sep 23;16(9):e0257686. doi: 10.1371/journal.pone.0257686 (PMC8459996; doi:10.1371/journal.pone.0257686)
Supplement: Attachment — Submitted filename: Response to reviewers-aug-16.pdf [file pone.0257686.s003.pdf]

List of responses:

For instance, the meaning of l and k in eq. (5) is still unexplained. The new sentence in lines 360-361 is unclear. What is c(5,95)? The following paragraph is also confusing (probably due to a grammatical error).

R: Definitions of l and k were extended in lines 262-277. The lines 360-365 were improved and explanation was extended. The definition of c(5,95) was also considered in the paragraph

I also, I would the authors to take into consideration the following points:
- When the intensity of the signal is negative, are the same values of l and k used? One would expect a different mechanism there, and therefore a different delay.

R: Regarding the combination of lags mentioned, we performed simulations with all possible combinations (with k=1,2,3; l=1,2,3, for X and Y respectively. Where k=l=3, the quantity of results within our sample with statistical significancy and were the ones presented in the paper. Information transference from both X->Y and Y->X (p-value=<0.1) was the largest in any of the scenarios:

a) ETE X->Y with p-value =<0.10 and ETE Y->X with p-value >0.10 , meaning that there is only information transfer from X to Y.

b) ETE X->Y with p-value >0.10 and ETE Y->X with p-value =<0.10 , meaning that there is only information transfer from Y to X.

c) ETE X->Y with p-value =<0.10 and ETE Y->X with p-value =<0.10 , meaning that there is information flux from both variables X to Y and viceversa.

To differentiate information flow, X of Y, in Eq. (9), we defined how the intensity of the signal would be measured:

$$\text{Intensity} = \text{ETE}(Y \to X)/[\text{ETE}(Y \to X) + \text{ETE}(X \to Y)] \qquad (9)$$

In this equation, if the information signal was greater from Y-> X, it would take a positive sign, meaning that there is a positive signal flowing from social media to stock prices, which is the main interest of our study. The contrary sign could be expected if the signal from X->Y was greater. This was performed exclusively to differentiate the direction of the signal in our table results.

In addition, that the effect of the news in the stock market 3 days after the news is released is a result confirmed that well other authors shared in different papers quoted (Johan Bollen, Huina Mao, Xiaojun Zeng. Twitter mood predicts the stockmarket. Journal of Computational Science.2011; 2:1–

Tahir M. Nisar, Man Yeung. Twitter as a tool for forecasting stock marketmovements: A short-window event study. The Journal of Finance and DataScience 4.2018:101-119)

- Fig. 6 does not show a clear-cut proof that negative signals have a stronger effect on the price than positive ones. This conclusion is therefore not sufficiently supported and should be weakened, or further analysis must be made.

Figure 6 was omitted for this review to avoid ambiguity in the presented results
